# Time-dependence of decontamination efficiency after a fallout of gamma-emitting radionuclides in suburban areas: a theoretical outlook on topsoil removal

**DOI:** 10.1038/s41598-022-25956-y

**Published:** 2022-12-15

**Authors:** Christopher L. Rääf, Mats Isaksson, Johan Martinsson, Robert Finck

**Affiliations:** 1 Medical Radiation Physics, Department of Translational Medicine, Lund University, 205 02 Malmö, Sweden; 2 Department of Medical Radiation Sciences, Institute of Clinical Sciences, Sahlgrenska Academy, University of Gothenburg, 41345 Gothenburg, Sweden

**Keywords:** Risk factors, Environmental impact, Applied physics, Nuclear waste

## Abstract

Decontamination of urban areas may be necessary in the case of extensive fallout of radioactive material after a nuclear accident, as removal of contaminated soil and vegetation will significantly reduce doses for the residents in an area affected by fallout. Experience from Japan shows that cleanup operations of urban areas may take years despite investment in ample resources. The time delay between the initial fallout and completion of the decontamination measures allows natural and physical processes to affect the results. The efficiency of the decontamination will therefore depend significantly on time. Radioecological modeling and computer simulation of urban topography with one-story houses were applied in this study to estimate action-influenced time-integrated dose reductions (*TDR*) of contaminated topsoil removal as a function of time after the fallout. Results indicate that the *TDR* decreases gradually after the fallout depending on the vertical migration rate of radiocesium and, to some extent, the initial ^134^Cs/^137^Cs ratio. Delaying the topsoil removal from 1 to 10 years will result in a *TDR* decrease by more than a factor of two. Removing the topsoil within one year after fallout results typically in an averted effective dose between 34 and 80 mSv per MBq m^−2^ deposition of ^137^Cs for residents in wooden houses. The corresponding values for residents in brick houses are about 50% lower due to higher shielding. Additional modeling is needed to estimate how age and sex influence the averted detriment to affected cohorts. In addition, more in-depth knowledge of how the efficiency of topsoil removal in practice compares with hypothetical models and the effect of incomplete removal of radiocesium is needed to improve calculations of *TDR* values.

## Introduction

An accidental release of radioactive elements from a ruptured nuclear reactor core with damaged safety barriers may contain volatile fission and neutron activation products, such as ^137^Cs and ^134^Cs. If released, these radionuclides can cause a considerable external radiation dose to humans when deposited on the ground in residential areas. Short-lived gamma-emitting radionuclides are also released, initially predominating the external dose rate. However, the more long-lived ^134^Cs (T_½_ = 2.06 y) and ^137^Cs (T_½_ = 30.0 y) will account for over 75% of the time-integrated external dose^1^. The surface deposition densities of ^137^Cs from the two large-scale nuclear accidents in Chernobyl, Ukraine, 1986, and Fukushima, Japan, 2011, reached regional average levels up 5 MBq m^−2^ or higher^2,3^. The cesium contamination with accompanying short-lived fission products results in 70 y time-integrated effective doses (including also the internal exposures from radioecological transfer of radiocesium) on the order of 100–500 mSv/(MBq m^−2^^137^Cs), depending on the effective ecological half-time of radiocesium, lifestyles and dietary habits and the level of protection^4,5^. Authorities ordered permanent or long-term evacuation of residents in areas with ^137^Cs deposition densities over 1 MBq m^−2^ in the former USSR and Japan.

After the Fukushima Dai-ichi accident in 2011, substantial areas (∼1300 km^2^) were subject to decontamination measures to enable resettlement of the evacuated population^6^. The reference dose value for resettlement was set to 20 mSv external dose the first year upon return, and residential areas with ^137^Cs surface deposition densities up to 3 MBq m^-2^ were part of the cleanup campaign^7,8^. The experience from this campaign showed that cleanup operations in residential areas may take several years to complete, even with the investment of considerable monetary and labor resources (e.g.,^7^). The efficiency of the decontamination measures will depend significantly on time, since natural and physical processers will gradually affect the nuclide composition and spatial distribution of the fallout (e.g.,^8,9^). The dose-reducing effect of early large-scale soil removal has been shown by^6^ and^10^, and its averted detriment was analyzed by^11^. The timing of this measure is critical, especially for younger age cohorts^11^.

The physical decay of the radionuclides reduces the dose rate from radiocesium deposited in the environment. In addition, natural processes redistribute the radionuclide deeper into the ground and remove part of it by runoff, furthering the dose rate reduction over time. The effective ecological half-life, measures the conjoined effects. The temporal trend of observed values in areas affected by radiocesium fallout often follow a bi-exponential pattern, with a short-term component of around 0.5 y (e.g.,^12^) and a long-term component that appears to vary substantially. The value of the latter has ranged from just 3.2 y in the Fukushima prefecture in Japan^13^ (although this data merges the physical decay of ^134^Cs and ^137^Cs) to 5 y in urban evacuated areas in Japan ^12^, 6.7 y in the vicinities of Gävle in Sweden^1^, and up to 15–20 y in rural Russian settlements^14^.

A more mechanistic convection–diffusion model for depicting the downward migration of radiocesium has been described by^15,16^. The effective diffusion coefficient, *D* (cm^2^ y^−1^), and the effective downward migration rate, *v* (cm y^−1^), determine the gradual migration of the element into the soil. A variant of this model was used by^17^ to extensively map the depth profile of radiocesium fallout in the ground in Sweden. Velasco^18^ expanded a convection–diffusion model to describe a bi-exponential time pattern of external dose rate above ground and presented a relationship between the parameters *D* and *v* and the corresponding short- and long-term ecological half-times of radiocesium. Redistribution of Cs contamination in the soil by either sedimentation of resuspended Cs or erosion processes affect the ambient dose rate above ground as time passes. As an example, in an area affected by large-scale deposition of radiocesium,^18^ theoretically demonstrated that a moderate sedimentation rate of 0.5 cm y^−1^ of contaminated soil particles in such an area would in some cases result in a slower decay rate of the ambient does rate than compared with no sedimentation. Given the prior use of the convection–diffusion model for Cs migration in soil, it has been found suitable for modelling how the external dose rate changes with time in contaminated areas, such as in gardens around residential buildings, as long as these are relatively undisturbed by mechanical actions during a temporary evacuation of the residents.

This study thus aims to theoretically predict the efficiency of topsoil removal actions when averting effective doses to residents in typical northern European settlements. It also seeks to understand how the ^134^Cs to ^137^Cs isotope ratio and local soil migration influence efficiency, both in terms of momentaneous dose reduction as well as the relative time-integrated dose reduction. The results are intended to be used for selecting appropriate soil removal strategies in connection with emergency preparedness management for future accidents. In this stage of the study, we have chosen not to specifically present the impact of the dose contributions from other urban surfaces, such as building roofs or road structures, since these need to be dealt with by means of other types of decontamination measures than topsoil removal.

## Materials and methods

### Modeling of an urban landscape and relative damping factors

**Figure 1:**
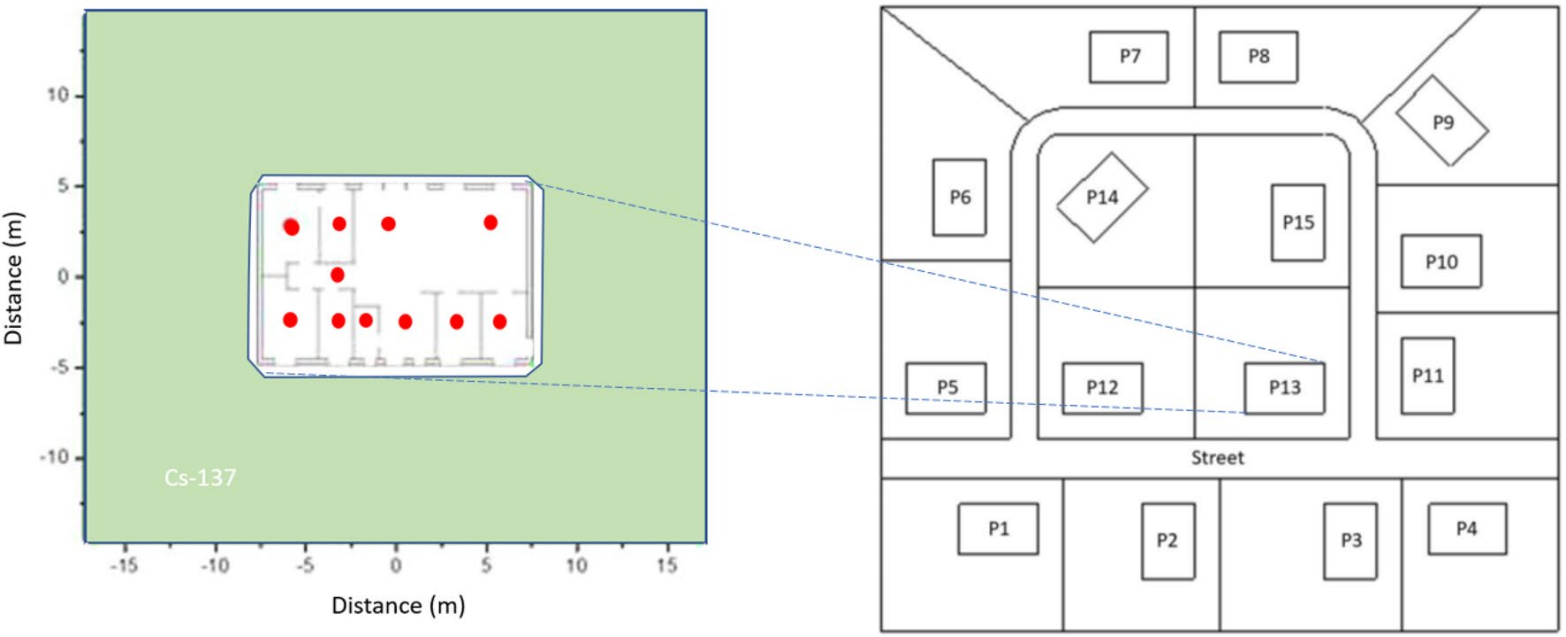
(Left) Schematic drawing of a modelled source geometry for a one-storey residential building (consisting either of brick or wood) surrounded by a rectangular deposition field of ^137^Cs extended 10 m from the building walls. Red dots represent 11 different observation points used by^8^ to compute air kerma rate. The average values of these air kerma rates are given in Table 1 and have been used in this study to derive the quantities relative damping factor, *RDF*_*in*_, and effective shielding factor, *ESF*. Details of the model can be found in^8^. (Right) Model of a suburban residential neighborhood as defined in^19^ and further used in^20^. The model includes 15 replicates of the residential building defined in^8^.

In a previous study by^8^ a computer model of two types of typical Northern European one-story building (one wooden and one brick) was made to simulate the air kerma values from a surrounding ^137^Cs deposition (Fig. 1:Left). The computer model was used to simulate the photon fluence from a deposition consisting of the gamma emitter ^137^Cs (*E*_*g*_ = 0.662 MeV) extending 10 m from the walls of the building. Air kerma rates at 11 different observation points inside the house (indicated as red dots in Fig. 1:Left) were calculated. Assuming zero depth penetration of the ^137^Cs deposition on the area surrounding a building made of wooden walls, the air kerma rate per unit surface activity of ^137^Cs inside the building was found to be 0.48 ± 0.20 (μGy h^−1^)/(MBq m^−2^) as an average for all 11 computation locations inside the house. For a brick house^8^ an average value of 0.26 ± 0.12 (μGy h^−1^)/(MBq m^−2^) was obtained. This simulation was then repeated but with the ^137^Cs deposition buried 2.5 and 5 cm into the soil (specified in the reference) to obtain the corresponding air kerma values in the observation points inside the building (Table 1).

**Table 1:** Indoor air kerma rate per ground deposition of ^137^Cs (± 1 SEM) from a surrounding surface deposition, as simulated by^8^ **, and the corresponding air kerma rate 1 m above an infinite surface deposition (taken from^21^ ), expressed in units of (μGy h^−1^)/(MBq m^−2^). SEM = standard deviation of the mean.

Burial depth*	Indoor air kerma rate for urban landscape (μGy h^−1^)/(MBq m^−2^)				Outdoor air kerma rate for infinite surface source, (μGy h^−1^)/(MBq m^−2^)
(cm)	Wooden houses		Brick houses		Free open surface
	11 points in single property	Whole suburban neighborhood	11 points in single property	Whole suburban neighborhood	
0	0.48 ± 0.052	0.93 ± 0.10	0.27 ± 0.030	0.52 ± 0.06	2.72
2.5	0.087 ± 0.011	N/A	0.045 ± 0.006	N/A	0.893
5	0.048 ± 0.006	N/A	0.025 ± 0.03	N/A	0.581

*Standard soil as defined by^22^.

**Disregards the contribution from the roofs of the residential buildings. According to^23^, with a fresh fallout, the roof contribution to the indoor air kerma rate, *K*_*air,in*_, can vary from 22 to 33% of the contribution from the surrounding gardens and streets, depending on whether the ^137^Cs fallout is wet or dry deposited.

Fifteen replicates of the residential building were then combined into a whole model of a suburban neighborhood (^19^ and ^23^complementary calculations). This simplistic neighborhood model thus consisted of a block of one-story houses, constructed of either wood or bricks, with designs typical for northern Europe and the northern temperate climate zone (for details refer to^8^) (see Fig. 1; Right). The total area of this landscape was 140·140 m = 19,600 m^2^, of which the entire street surface was 1961 m^2^ and total roof area (projected to the surface plane) was 2250 m^2^, resulting in a remaining total garden area of 15,389 m^2^. The simulated ^137^Cs deposition was distributed over the garden areas and the streets, and used to compute air kerma dose rates in observation points located in each of the buildings.

In the above study^8^ the relative fluence contribution to the indoor air kerma rate from a homogeneous zero depth surface contamination of ^137^Cs with an infinite extent was also computed. On average, 51.2% of the contribution originated from deposition outside a single property around a wooden house. The corresponding value for a brick house was 56.3%. In the present study we have merged the results from^8^ with^19^ to obtain estimates of the average indoor air kerma rate inside a wooden and brick building, positioned in the middle block of the modelled neighborhood (as depicted in Fig. 1: Right) by assum that it surrounded by an infinite surface of ^137^Cs deposition. The estimates are presented in Table 1, where the average indoor air kerma rate in a wooden house is estimated to be 0.93 (μGy h^−1^)/(MBq m^−2^) and somewhat lower, 0.52 (μGy h^−1^)/(MBq m^−2^), in a one-storey brick building.

As a comparison, for an infinite plane surface (free open surface) with a homogeneous deposition of ^137^Cs, ICRP^21^ gives an air kerma rate per deposition of 2.72 (μGy h^−1^)/(MBq m^−2^). In the present study, the ratio of air kerma rates between the indoor values and the corresponding value 1 m above ground for an infinite contaminated ground surface (as taken from^21^) forms a measure of the shielding properties of the buildings, here denoted as the effective shielding factor (*ESF*). The *ESF* for a single wooden building in the modeled landscape^8^, will thus be 0.93/2.72 = 0.35 ± 0.05 (± 1 standard deviation of the mean), and the corresponding value for a brick house will be 0.17 ± 0.03.

To account for the gradually increasing penetration of ^137^Cs into the ground with time, the air kerma rate 1 m above ground for burial depths 2.5 and 5 cm was calculated for a single wooden house and a brick house^8^. The depth distribution of ^137^Cs was simplified to a plane geometry covered with an inactive layer of soil of thickness equal to the burial depth. The surface source extended over a limited area of 1050 m^2^ around a wooden or brick building. The simulations used a soil composition model taken from^22^. A relative damping factor (*RDF*_*in*_) was then defined as the indoor kerma rate for the ^137^Cs burial depths (2.5 and 5 cm, respectively) normalized to the indoor kerma rate for the zero-penetration surface source. The calculated *RDF*_*in*_ showed no significant difference between the wooden and brick houses (0.18 ± 0.03 vs. 0.17 ± 0.03 at 2.5 cm burial depth, and 0.099 ± 0.02 vs. 0.094 ± 0.02 for 5 cm burial depth, respectively).

A multi-exponential function of soil depth, x, was then fitted to the calculated *RDF*_*in*_ values for the wooden and brick houses at 0, 2.5, and 5 cm burial depths to obtain a continuous expression given in Eq. (1):

1 $$RDFin\left( x \right) = 0.5e^{{ - \left( {\frac{{{\text{ln}}2}}{3}} \right) \cdot x}} + 0.25e^{{ - \left( {\frac{{{\text{ln}}2}}{{0.2}}} \right) \cdot {\text{x}}}} \cdot + 0.25e^{{ - \left( {\frac{{{\text{ln}}2}}{{0.92}}} \right)}}$$

**Figure 2:**
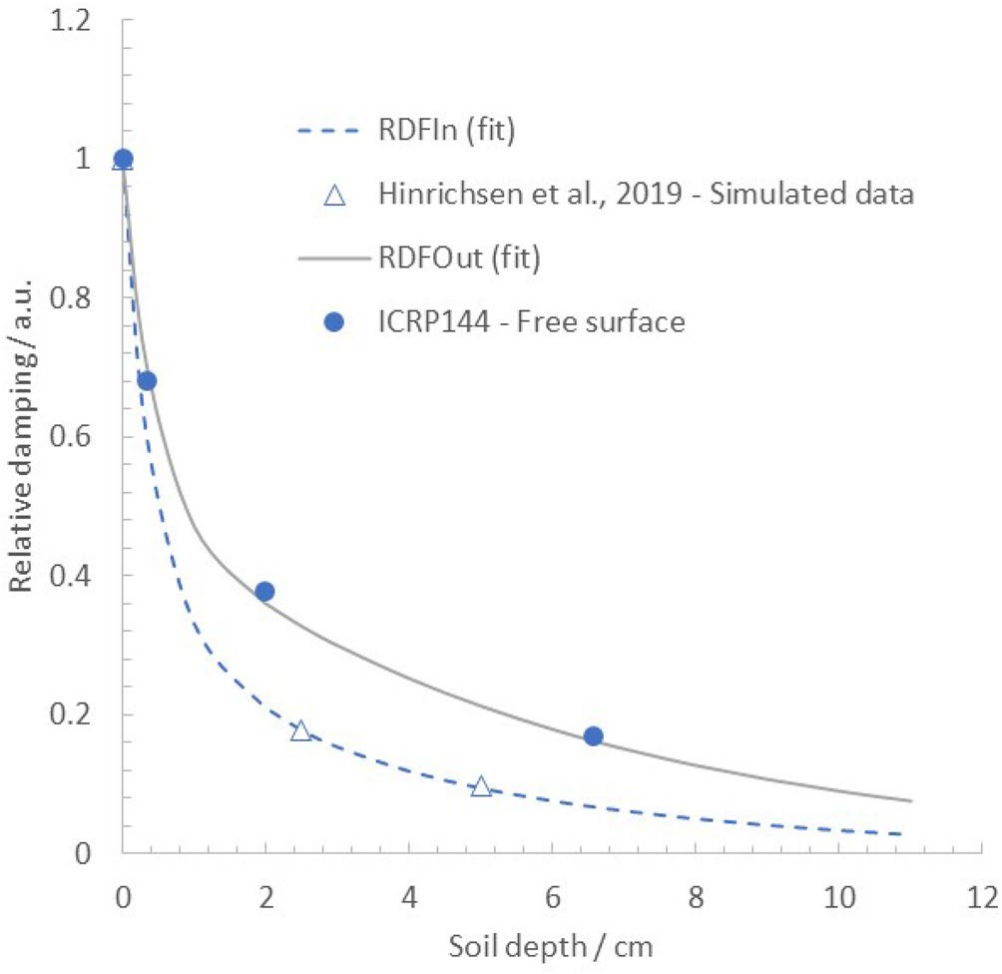
Relative damping factor, *RDF (RDF*_*in*_ = indoor occupancy and *RDF*_*out*_ = outdoor occupancy*)*, of air kerma rate 1 m above ground level as a function of burial depth of ^137^Cs deposition obtained from fitting bi-exponential decay functions to simulated data provided from^19^ and^21^.

Note that *RDF*_*in*_ thus describes the indoor air kerma rate from a 1050 m^2^ surrounding property contaminated with ^137^Cs as a function of source depth in the ground relative to that with zero penetration (x = 0 cm) (Fig. 2). Moreover,^21^ presents simulated air kerma rate to deposition density conversion factors for infinite ^137^Cs planar sources at four different soil burial depths, expressed in mass depth (g cm^−2^). Assuming a negligible difference in the atomic composition of the simulated soil between^22^ and^21^, the ICRP conversion factors could be plotted against the nominal depth *x* (cm) in soil of density 1.5 g cm^−3^. Thus, the conversion factor for a surface contamination (corresponding to a mass depth 0 g cm^−2^) was assigned to *RDF*_*out*_ (x = 0 cm), and the corresponding conversion factors for mass depths 0.5, 3, and 10 g cm^−2^ were assigned to *RDF*_*out*_ (x = 0.33 cm), *RDF*_*out*_ (x = 2.0 cm), and *RDF*_*out*_ (x = 6.6 cm), respectively. A corresponding curve fit to *RDF*_*out*_ could then be computed, giving the multi-exponential function in Eq. (2) (plotted in Fig. 2).

2 $$RDFout\left( x \right) = 0.5e^{{\left[ { - {\text{ }}\left( {\frac{{{\text{ln}}2}}{{2.3}}} \right) \cdot x} \right]}} + 0.5e^{{ - \left( {\frac{{{\text{ln}}2}}{{0.17}}} \right){\text{x}}}}$$

In the continued assessment of the indoor and outdoor external exposure of residents in the modelled suburban neighborhood, *RDF*_*in*_ is here used as a conservative estimate for the contribution to the effective dose rate from the radiocesium contents in various soil layers surrounding the modeled wooden and brick houses. It can be shown mathematically (see Appendix 1) that the *RDF* for indoor air kerma rates in a real urban landscape will decrease more rapidly than *RDF*_*in*_ fitted to data from^8^, since the surrounding buildings will provide some shielding when considering the kerma rate contribution from deeper soil layers. Likewise, the fitted function for the *RDF*_*out*_ adapted from the ICRP^21^ source geometries will also result in conservative air kerma rate estimates 1 m above ground for outdoor locations in an urban landscape. This is because the presence of sheltering objects, such as vegetation and surrounding buildings, will provide additional damping of the contribution from deeper layers of radiocesium.

### Modeling of air kerma rate and effective dose rate above ground as a function of radiocesium migration into the soil with time

**Figure 3:**
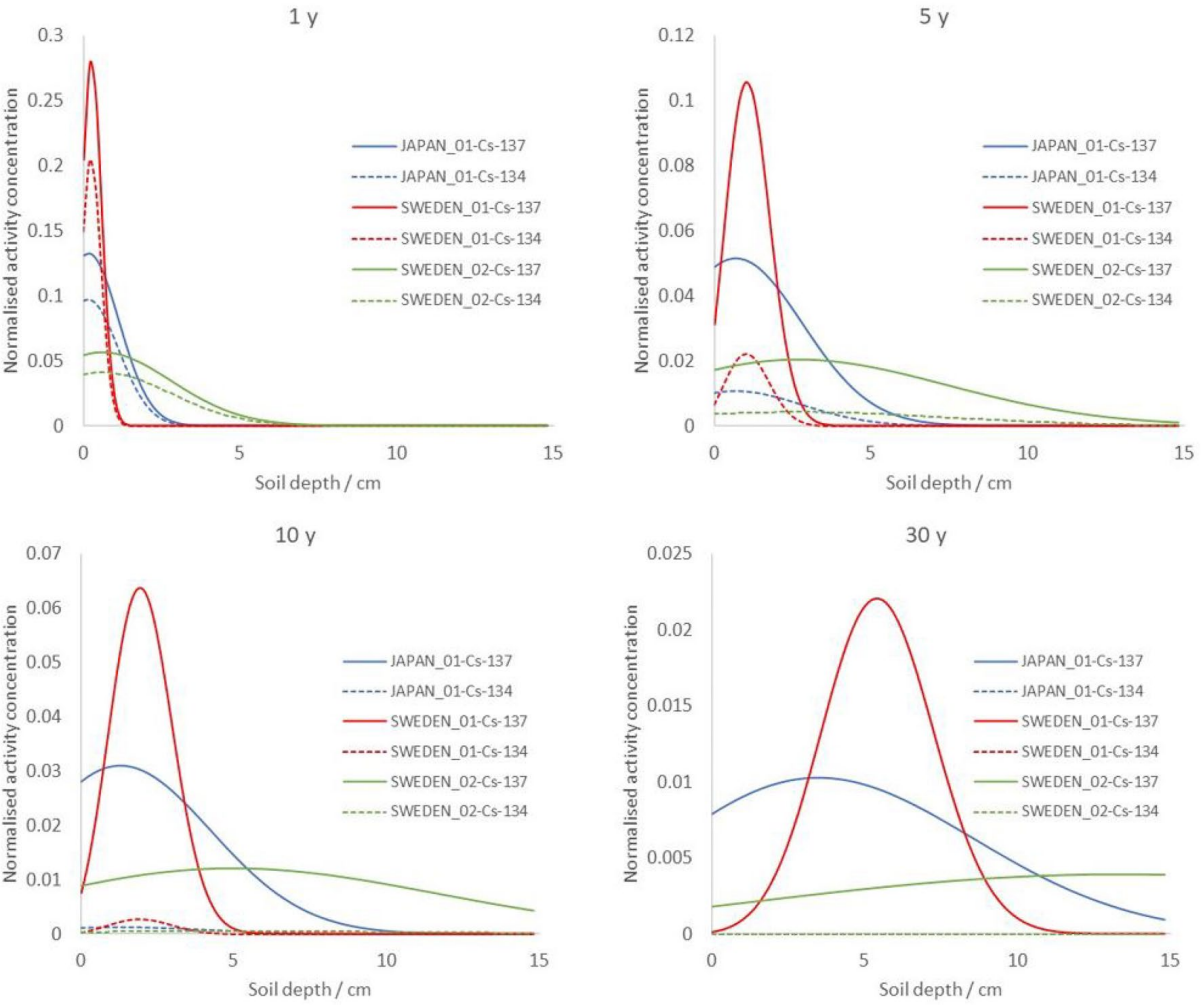
Soil depth profile for ^137^Cs and ^134^Cs (with initial ^134^Cs:^137^Cs ratio of 1) for three different settings of vertical soil migration parameters after 1 y (top left), 5 y (top right), 10 y (bottom left), and 30 y (bottom right) after fallout (see also Table 2). The profiles are calculated for a soil with a density of 1.5 g cm^−3^, and are normalized to the cumulative soil concentration at *t* = 0 for the respective Cs isotopes.

The vertical transport of radioactive contaminants in soil can be described as a function of time and vertical soil depth, *x* (cm), by a convection–diffusion model, as suggested by[^24^, ^25^] (Eq. 3):

3 $$C\left( {x,t} \right) = C_{0} \cdot e^{{ - ln(2) \cdot \frac{t}{{T_{{phys}} }}}} \cdot \left[ {\left( {\frac{1}{{\sqrt {\pi \cdot D \cdot t} }}} \right) \cdot e^{{ - \frac{{(x - v \cdot t)^{2} }}{{4D \cdot t}}}} - \left( {\frac{v}{{2D}}} \right) \cdot e^{{\left( {\frac{v}{D} \cdot x} \right)}} \cdot erfc\left( {\frac{{x \cdot t + v}}{{2\sqrt {D \cdot t} }}} \right)} \right]$$

where C_0_ is the initial contaminant concentration (Bq cm^−3^), *T*_*phys*_ is the physical half-life of the radiocesium isotope (*T*_*phys,Cs-137*_ = 30.0 y and *T*_*phys,Cs-134*_ = 2.06 y), *D* is the effective diffusion coefficient (cm^2^ y^−1^), and *v* is the convective velocity (cm y^-1^). The expression in Eq. (3) can then be normalized to ${\int }_{0}^{\infty }C\left(x,t\right)dx$=1 by numerical integration of C(x,t) with depth *x* at selected times *t* and decay corrected with the physical half-lives of ^137^Cs and ^134^Cs, respectively, to obtain curves illustrated in Fig. 3.

Three parameter settings for the constants *D* and *v* in Eq. (3) were selected in this study to represent different soil migration scenarios. The first setting is based on sample data collected outside Fukushima Dai-ichi in April 2016, as presented in^26^, where, on average, about 70% of the radiocasium was confined to the top 2 cm layer, 25% in the 2 to 4 cm layer, and the remaining fraction in the 4–6 cm layer. This setting has here been denoted JAPAN_01. The second parameter setting is based on the radiocesium concentration profile observed in a location called Stenungsund in Sweden, as reported by^17^, and is here denoted SWEDEN_01. A third setting has been selected based on the upper end value of observed *D* and values in Sweden as presented in^17^. An overview of the parameter settings is given in Table 2.

The effective dose rate contribution above ground from the radiocesium at different depths, *x*, is estimated by numerically convoluting the normalized concentration curves, *C*_*norm*_(x,*t*) = C(x,t)/ ${\int }_{0}^{\infty }C\left(x,t\right)dx$, by the *RDF*(x) obtained previously for indoor and outdoor exposure (here approximated as a free surface), respectively^21^ (Eq. 4).

4 $$d{\dot{E}}_{Cs,\frac{in}{out}}/dx(x,t)={{ESF}_{in/out}\cdot C}_{norm}(x,t)\cdot {RDF}_{in/out}(x)\cdot {e}_{ICRP144}({E}_{\gamma },x=0)$$

The *RDF* refers to either indoor occupancy in a wooden or brick house, as specified in Table 1, or to outdoor exposure. The outdoor space is conservatively assumed to be a free open area with an infinite uniform surface distribution of radiocesium. The coefficient ***e***_*ICRP144*_ (*E*_*γ*_,x = 0) refers to the sum of tabulated conversion factors from ^137m^Ba and ^137^Cs valid for an effective dose rate to an adult 1 m above an infinite and shallow (penetration depth at 0.0 g cm^2^) surface distribution of ^137^Cs on the ground. For ^134^Cs, the corresponding tabulated value was used^21^. *ESF* is assumed to exhibit the same ratio for the effective dose rate between indoor and outdoor exposure as the corresponding ratio for *K*_*air*_. The coefficient ***e***_*ICRP144,Cs*_ (nSv h^−1^ Bq^−1^ m^−2^) will assume the value of 5.22 × 10^−3^ for ^134^Cs and 2.13 × 10^−3^ for the sum of ^137m^Ba and ^137^Cs. For outdoor occupancy, *ESF*_*out*_ is assigned a value of 1 for computation of ${\dot{E}}_{Cs,out}(t)$ over a uniform free surface. When integrated for all depths *x*, the effective dose rate 1 m above ground can be expressed as (Eq. 5):

5 $${\dot{E}}_{\mathrm{Cs},\frac{\mathrm{in}}{\mathrm{out}}}(\mathrm{t})={\int }_{0}^{\mathrm{x}}{{ESF}_{in/out}\cdot C}_{norm}(x,t)\cdot {RDF}_{in/out}(x)\cdot {e}_{ICRP144}({E}_{\gamma },x=0)\mathrm{dx}$$

where the left-hand side of Eq. (5) now will be a function solely of time when integrated over all soil depths *x*. The effective dose rate for the normalized concentration profiles of JAPAN_01, SWEDEN_01 and SWEDEN_02 can then be estimated for indoor and outdoor occupancy in the modelled neighborhood without any cleanup measures.The settings in Table 2 furthermore correspond to different effective ecological half-times of the radiocesium attributed contribution to the external dose rate 1 m above ground, and can be deduced by fitting time dependent exponential functions to the derive effective dose rate in Eq. 5 for either *RDF*_*in*_ (indoor occupancy) or *RDF*_*out*_ (outdoor occupancy).

To compute the time-integrated effective dose to an adult in the modelled neighborhood, *E*(50 y), the expression in Eq. (5), has been time-integrated to t = 50 y, giving the 50-year time-integrated effective dose to residents without soil removal procedures, referred to here as the “unmitigated dose”.

### Modeling action-influenced initial and time-integrated dose and radiation-risk reduction by soil removal

Using convection–diffusion equations, it is possible to numerically compute how indoor and outdoor effective doses to people in the modeled area are affected by soil removal, with or without refilling of clean soil. In this study we have assessed the reduction of external dose from soil removal without refilling, which was the common practice in residential areas affected by the Fukushima accident^6^. Since radioceasium from accidental nuclear power plant releases will contain both ^137^Cs and ^134^Cs, it is necessary to include the contribution from both these isotopes in Eq. (3). A simplification is made to facilitate the computations by setting the ratio between the dose coefficients ***e***_*ICRP144*_ for ^134^Cs and ^137^Cs to be constant (= 2.48) with the burial depth of radiocesium. According to ICRP^21^, these ratios fluctuate between 2.48 and 2.57 for the burial mass depths applied in our calculation. As mentioned previously, the dose conversion factor between ^137^Cs deposition on the ground surface (burial depth of 0 g cm^2^) and effective dose rate to an unshielded adult, ***e***_*ICRP144,Cs-137*_ (*x* = 0), is 2.13 µSv h^−1^/MBq m^−2^, which corresponds to an annual dose rate of 18.5 mSv y^−1^/MBq m^−2^.

Furthermore, it is assumed that the normalized concentration profiles, *C*(*x*,*t*), for ^137^Cs and ^134^Cs are equal due to negligible isotope effects in the elemental transport in soil. With these assumptions, a simplistic expression of the summed effective dose rate contribution from ^134^Cs and ^137^Cs 1 m above ground inside a wooden or brick house at a specific time *t* after the fallout—and without mitigation—can be expressed as follows (Eq. 6):

6 $${\dot{{E}}}_{{Cs},{in}}({t})={\int }_{0}^{{x}=50{ cm}}{{{ESF}}_{{in}}\cdot {C}}_{{norm}}\left({x},{t}\right)\cdot (1+\left({{\mathbf{e}}_{{ICRP}144,{Cs}-134}}/{{\mathbf{e}}_{{ICRP}144,{Cs}-137}}\right)\cdot {{e}}^{-\left(\frac{\mathrm{ln}2}{{\mathrm{T}}_{\mathrm{phys},\mathrm{Cs}-134}}-\frac{\mathrm{ln}2}{{\mathrm{T}}_{\mathrm{phys},{Cs}-137}}\right)}){\cdot {RDF}}_{{in}}({x})\cdot {\mathbf{e}}_{{ICRP}144,Cs-137}({{E}}_{\upgamma },{x}=0){dx}$$

The upper integral limit was set to about 50 cm, since the contribution to the effective dose rate 1 m above ground will be damped according to the *RDF*_*in*_ (and *RDF*_*out*_*)* factor to less than 0.1% of that from the contamination in the surface layer of the soil.

For a topsoil removal of a *d* cm thick layer in the area around a residential house in the modeled landscape performed at time *t* = *t*_*cleanup*_ after the radiocesium deposition, the *RDF* function (both *RDF*_*in*_ and *RDF*_*out*_) can be approximated as *RDF* (*x-d*), provided *d* <  < 1 m. The remaining effective dose rate per unit ^137^Cs deposition (mSv (MBq m^−2^)^−1^) at 1 m above ground level inside the building can then be expressed as

7 $${\dot{{E}}}_{{Cs},{in},{cleanup}}\left({t}={{t}}_{{cleanup}}\right)={\int }_{{x}={d}}^{{{x}}^{{^{\prime}}}=50{ cm}}{{{ESF}}_{{in}}\cdot {C}}_{{norm,rem}}\left({x}>{d},{{t}}_{{cleanup}}\right ) \cdot (1+\left({{\mathbf{e}}_{{ICRP}144,{Cs}-134}}/{{\mathbf{e}}_{{ICRP}144,{Cs}-137}}\right)\cdot {{e}}^{-\left(\frac{\mathrm{ln}2}{{\mathrm{T}}_{\mathrm{phys},\mathrm{Cs}-134}}-\frac{\mathrm{ln}2}{{\mathrm{T}}_{\mathrm{phys},{Cs}-137}}\right)}){\cdot {RDF}}_{{in}}({x}-{d})\cdot {\mathbf{e}}_{{ICRP}144,Cs-137}({{E}}_{\upgamma },{x}=0){dx}$$

where *C*_*norm,reml*_(*x*,*t*) is the soil concentration profile after removal of *d* cm given by

8 $${{C}}_{{norm},{rem}}\left({x},{t}\right)=0 \mathrm{ for x}<{d };{=C}_{norm,rem}\left(x,t\right){ for x}\ge d$$

The ratio between Eq. (7) and Eq. (6), here denoted as action-influenced initial dose rate reduction (*IDR* = ${\dot{E}}_{Cs,in,cleanup}\left(t={t}_{cleanup}\right)/{\dot{E}}_{Cs,in}(t={t}_{cleanup})$), is a dimensionless number that indicates the relative dose rate reduction just after removing a topsoil layer of depth *x* in relation to that dose rate at the same time without this action. *IDR* is a function of the soil migration parameters giving *C*(*x*,*t*), the removed topsoil depth *d*, and the time *t* after the initial fallout. In this study, *IDR*(*C*,*x*,*t*) was calculated numerically for the three soil migration types given in Table 2, topsoil removal thicknesses *d* ranging from 1 to 5 cm, and implemented at eight different times t = 1 to 30 years after the fallout. The *IDR* function allows a prediction of the efficiency in the dose-rate-reduction effect from a specified soil-removal depth as a function of time after the fallout. This contrasts with previously assumed, fixed time-independent cleanup efficiencies of 50% and 90%^11^.

In order to model how the effective dose rate above the remediated soil after *t*_*cleanup*_ will depend on time, some simplifications have to be made. In the layers larger than *x* = *d* after the soil removal, there will be a remaining fraction of the ^137^Cs ground deposition (dimensionless), *δ*_*rem*_, given by Eq. (9):

9 $${\delta }_{rem}(d,{t}_{clean-up})=\frac{{\int }_{d}^{\infty }{C}_{norm}\left(x,{t}_{cleanup}\right)dx}{{\int }_{0}^{\infty }{C}_{norm}\left(x,{t}_{cleanup}\right)dx}$$

Two simplifications enable numerical computations of the time pattern in the dose contribution *t* > *t*_*cleanup*_. The first assumption is that, at *t* = *t*_*cleanup*_, the depth distribution of the remaining fraction, *δ*_*rem*_, is mathematically confined to a plane (single layer) at a burial depth *d*_*rem*_ (cm). In turn, *d*_*rem*_ is computed as the *fluence*-weighted mean value of the remaining soil concentration profile, *C*_*norm,rem*_(*x* > *d*, *t* = *t*_*cleanup*_), at the time right before *t*_*cleanup*_. The second simplification is that the remnant activity, located at depth *d*_*rem*_, will continue to behave according to the convection–diffusion model applied to the untouched soil layers. Given these simplifications, Eq. (7) can be rewritten so that the time dependence of the effective dose contribution from *δ*_*rem*_ after the cleanup (*t* > *t*_*cleanup*_) can be approximated according to Eq. (10):

10 $${\dot{{E}}}_{{Cs},{in},{cleanup}}\left({t}>{{t}}_{{cleanup}}\right) = \int\limits_{{{{x}}^{\prime } = 0}}^{{{{x}}^{\prime } = 50\;{\text{cm}}}} {{{ESF}}_{{{{in}}}} \cdot {{RDF}}_{{{{in}}}} \left( {{{x}}^{\prime } = {{d}}_{{{{rem}}}} } \right) \cdot {{RDF}}_{{{{in}}}} \left( {{{x}}^{\prime } \ge {{d}}_{{{{rem}}}} } \right) \cdot {\updelta }_{{{{rem}}}} \left( {{{t}} > {{t}}_{{{{cleanup}}}} } \right)} \cdot {{C}}_{{{{norm}},{{rem}},{{cleanup}}}} \left( {{{x}}^{\prime } ,{{t}} > {{t}}_{{{{cleanup}}}} } \right) \cdot \left( {1 + \left( {\frac{{{\mathbf{e}}_{{{\text{ICRP}}144,{\text{Cs}} - 134}} }}{{{\mathbf{e}}_{{{\text{ICRP}}144,{\text{Cs}} - 137}} }}} \right){{e}}^{{ - \left( {\frac{{{\text{ln}}2}}{{{\text{T}}_{{{\text{phys}},{\text{Cs}} - 134}} }} - \frac{{{\text{ln}}2}}{{{\text{T}}_{{{\text{phys}},{\text{Cs}} - 137}} }}} \right)}} } \right) \cdot {{RDF}}_{{{{in}}}} \left( {{{x}}^{\prime } } \right) \cdot {\mathbf{e}}_{{{{ICRP}}144,Cs-137}} \left( {{{E}}_{{\upgamma }} ,{{x}} = 0} \right){{dx}}$$

where *C*_*norm,rem,cleanup*_(*x*, *t* > *t*_*cleanup*_) is the ^137^Cs concentration at depth *x* along the depth of the remediated ground and normalized to the total activity of the remnant deposition, *δ*_*rem*_(*t* > *t*_*cleanup*_), at time *t* after *t*_*cleanup*_. *RDF*(*d*_*rem*_) is the damping factor of the remaining fraction of radiocesium attributed to the attenuation of the topsoil down to *d*_*rem*_. In addition to shielding effects of the remaining soil layer of the “new” single layer of remnant cesium after cleanup, the gradual migration of the remnant deposition will result in additional shielding effects for the layers *x* > *d*_*rem*_, which is accounted for by the relative damping factor *RDF*(*x* > *d*_*rem*_).

The action-influenced time-integrated dose reduction, *TDR*, of the soil removal in terms of averted effective dose attributed to the cleanup measure at time *t* = *t*_*cleanup*_ can be obtained by the difference in the time integration of the external dose from the ground deposition of a specified fallout of radionuclides, with and without decontamination actions, according to the following (Eq. 11):

11 $${TDR}\left({{t}}_{{cleanup}}\right)=\frac{\left\{{\int }_{0}^{\mathrm{t}=50\mathrm{ y}}\frac{\mathrm{d}{\dot{\mathrm{E}}}_{\mathrm{Cs},\mathrm{in}/\mathrm{out}}}{\mathrm{dx}}\left(\mathrm{t}\right)\mathrm{dt}-({\int }_{0}^{{\mathrm{t}}_{\mathrm{cleanup}}}\frac{\mathrm{d}{\dot{\mathrm{E}}}_{\mathrm{Cs},\frac{\mathrm{in}}{\mathrm{out}}}}{\mathrm{dx}}\left(\mathrm{t}\right)\mathrm{dt}+{\int }_{{\mathrm{t}}_{\mathrm{cleanup}}}^{\mathrm{t}=50\mathrm{ y}}\frac{\mathrm{d}{\dot{\mathrm{E}}}_{\mathrm{Cs},\mathrm{in}/\mathrm{out},\mathrm{clean}-\mathrm{up}}}{\mathrm{dx}}\left(\mathrm{t}\right)\mathrm{dt})\right\}}{{\int }_{0}^{\mathrm{t}=50\mathrm{y}}\frac{\mathrm{d}{\dot{\mathrm{E}}}_{\mathrm{Cs},\mathrm{in}/\mathrm{out}}}{\mathrm{dx}}\left(\mathrm{t}\right)\mathrm{dt}}$$

This study has only considered doses integrated over 50 y after the fallout to reference adult members of the public who are assumed to be evacuated from the area until clean-up is completed. When considering lifetime risks, the dose and risk calculation should also include the age and gender distribution of the population. This would, however, require a more detailed analysis of organ doses for the varying depth distribution of radiocesium, which is beyond the scope of this work. Furthermore, for the calculations of the air kerma and effective dose rate per unit initial ^137^Cs deposition after soil removal, a value of *d* = 5 cm has been used in this study in the numerical computations of Eqs. (7–10) to estimate the *TDR*. The choice of 5 cm was based on the depth commonly used in situ in Japan^6^.

## Results and discussion

### Action-influenced initial-dose reduction from topsoil removal in a residential area as a function of time for cleanup

**Figure 4:**
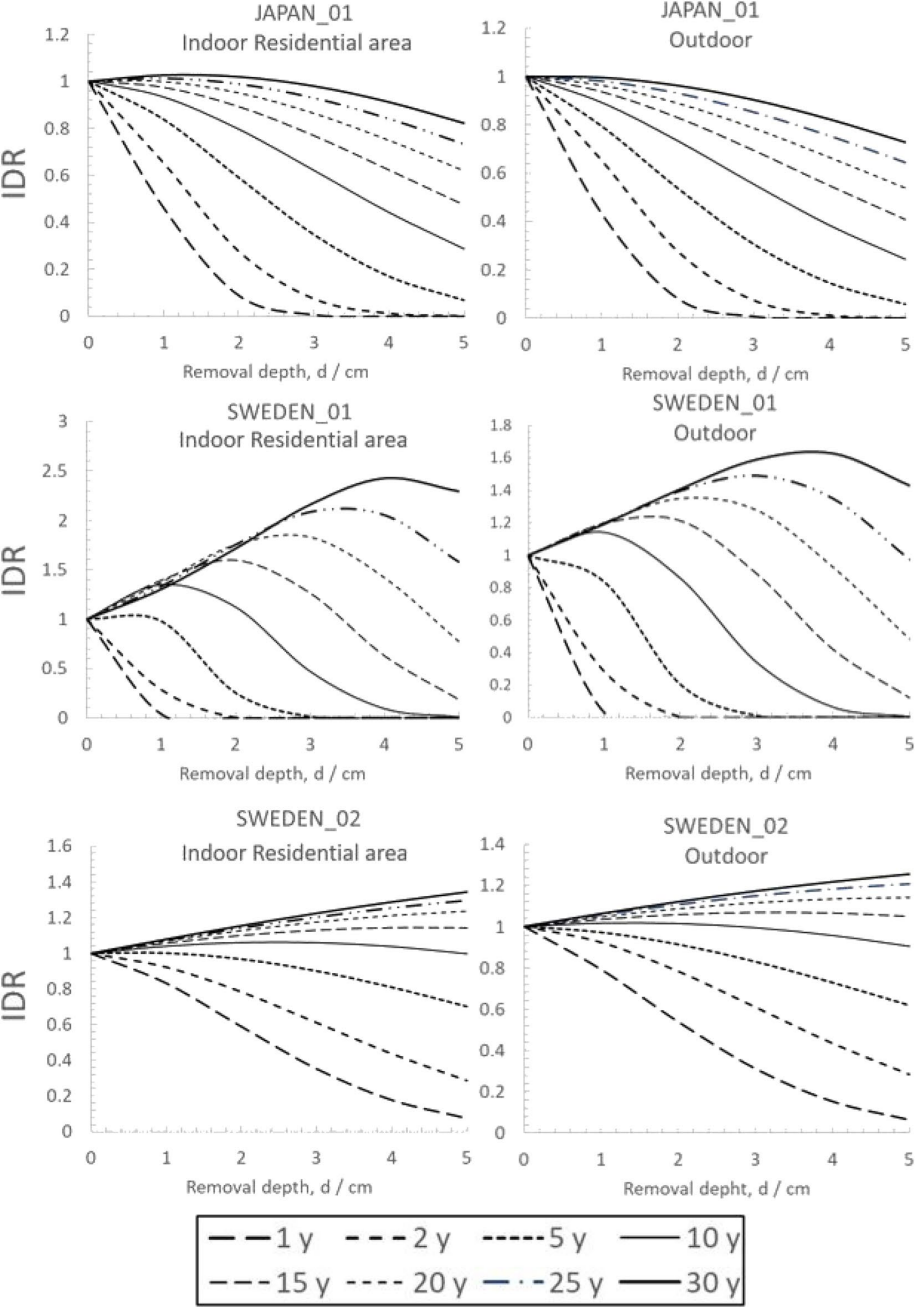
Action-influenced initial relative-dose rate reduction, *IDR* (in relative units), as a function of topsoil depth removal (without refill) in a suburban area when conducted at a certain time, *t*_*cleanup*_, after fallout. Plots are given for a selection of different *t*_*cleanup*_ between 1 and 30 y. Plots (left) refer to indoor occupancy in a typical northern European wooden or brick building and (right) outdoor exposure in an open area^21^. Note that there is no significant difference in *RDF* between brick and wooden buildings (see Table 1). Therefore, the plots (left) will be the same for the two building types. Three sets of soil migration parameters are represented: JAPAN_01 (top frames), SWEDEN_01 (middle frames), and SWEDEN_02 (bottom frames).

The models described in the previous section were used to compute the estimated time pattern of external dose contribution to inhabitants in one-story residential buildings from ground contamination without any soil removal. Figure 4 presents the calculated action-influenced dose rate reduction, *IDR*(*t*_*cleanup*_), by soil removal for various removal thicknesses between 1 to 5 cm and for different choices of delay times to a single cleanup measure after fallout, *t*_*cleanup*_. Note that the ^134^Cs/^137^Cs ratio will not affect *IDR*(*t*_*cleanup*_), because the isotopes will have the same physical and chemical properties in the soil and the mean gamma energy emitted from the isotopes is approximately the same.

Figure 4 shows the great influence that the soil migration settings have on the outcome of a single cleanup measure in terms of *IDR* for a certain depth of soil removal. For all three soil migration settings, a 5 cm removal depth will result in an *IDR* less than 0.4 provided the soil removal is done within 5 y after the fallout. However, delaying the cleanup further will result in substantially less reductions of external dose (meaning that *IDR* will assume values closer to 1), especially for the profile SWEDEN_02, mainly due to its high diffusion value, *D* = 2.63 cm^2^ y^−1^, representing a situation where the radioceasium will be relatively confined around the depths between 5 to 10 cm. A strategy would then be to remove also the soil deeper than 5 cm, or refilling the surface with uncontaminated soil, but both solutions will implicate higher amount of costly soil transportation and distribution.

**Figure 5:**
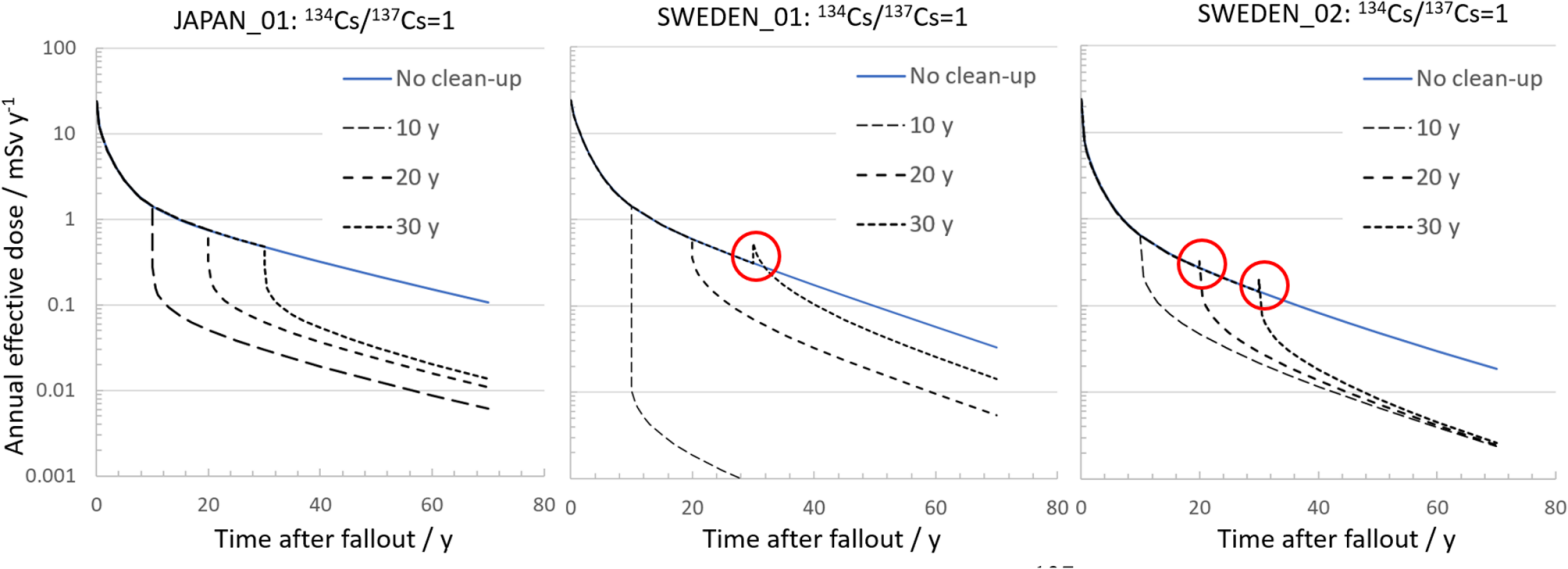
Time pattern of external effective dose rate per unit ^137^Cs deposition ((mSv y^−1^)/(MBq m^−2 137^Cs) for indoor occupancy in residential wooden houses for the three different soil migration settings and for a ^134^Cs/^137^Cs ratio of 1, for various time delays to cleanup, *t*_*cleanup*_ with 5 cm topsoil removal. The cases with tardy scraping effects are highlighted with red circles.

A surprising fact is observed. For two of the three soil migration settings (SWEDEN_01 and SWEDEN_02), delaying topsoil removal for ten years or more may cause a reverse in the *IDR* (meaning that *IDR* will become larger than 1) and the procedure may instead *increase* the external dose rate (Fig. 4). The effect is here referred to as a “tardy scraping effect,” since it arises from removing the protective shield provided by the topsoil layer at a later time when much of the radioactive material has migrated into the ground below the scraping level (Fig. 3). The tardy scraping effect can also be illustrated by plotting the time series of the estimated external dose rate as a function of time for various cleanup delay times (Fig. 5).

Empirical findings from Japan show that, on average, the *IDR*, as measured close to the ground surface*,* around the time of cleanup (ranging between 1 and 5 y) is 100%-58% = 42%^6,27^ for an average topsoil removal of 3.7 cm. Given that this average value is based on collimated near-surface measurements, it can be compared with the predicted remnant radiocesium fraction at *t*_*cleanup*_ = 5 y after 5 topsoil removal, ${\delta }_{rem}$, of the depth profile characterized by JAPAN_01 (Table 2), which is only 22% (Fig. 4:top left). The somewhat lower value of ${\delta }_{rem}$ is thus not fully compatible with the real outcome of the cleanup measures in Japan.

At least three plausible explanations can be hypothesized regarding the found discrepancy between the theoretical and actual attained decontamination effects from topsoil removal observed in Japan after the Fukushima accident. One could be that the convection–diffusion model used in the present study does not consider the additional deposition of contaminated vegetation or general sedimentation. However, if the *IDR* is measured with a collimated detector on a reasonably open surface, this addition should not influence the measured *IDR* very much. Another explanation could be that the actual soil profiles at the decontaminated sites varied substantially from that observed by^26^. If instead, assuming the soil depth profile SWEDEN_02 represents the remediated areas in Japan, then a 42% relative dose reduction becomes more realistic after five years. A third explanation could be that the mechanical processes in shuffling away soil masses induce inadvertent soil mixing of deeper layers, leading to less of the contaminated surface layer being removed.

The results indicate that the choice of removal depth of topsoil in an area aimed to be decontaminated should be adapted to the activity concentration profile *C*(*x*,*t*). For cleanup measures carried out several decades after the fallout, soil profile investigations are needed to select an appropriate removal depth that avoids the tardy scraping effect, as shown in, e.g., Fig. 5.

### Action-influenced time-integrated dose reduction from topsoil removal in a residential area

As a reference case with no evacuation and no cleanup, the radiocesium contribution to the projected unmitigated effective dose over 50 y to an adult staying indoors in a wooden house is given in Table 3. This unmitigated dose contribution will vary between 34 and 86 mSv per MBq m^−2 137^Cs, depending on the three types of soil migration settings described in Table 2. This is valid for ^134^Cs/^137^Cs ratios between 0.56 (as in the Chernobyl fallout) and 1.47 (as in Swedish NPP inventories). The action-influenced time-integrated dose reduction, *TDR*(*t*_*cleanup*_), by 5 cm topsoil removal over 50 y for the three different soil migration settings is presented in Table 3. It is assumed that the inhabitants are evacuated from time *t* = 0 to *t* = 1 y = *t*_*cleanup*_, and hence the averted effective dose after 1 y refers to the dose averted by evacuation in combination with 5 cm topsoil removal.

**Table 2:** Parameter settings for the three types of soil (JAPAN_01, SWEDEN_01 and SWEDEN_02) in terms of radiocesium migration parameters *D* and *v* in Eq. (3).

	JAPAN_01*	SWEDEN_01**	SWEDEN_02***
*D* (cm^2^ y^-1^)	0.5	0.06	2.63
*v* (cm y^-1^)	0.08	0.17	0.35

*Based on soil sample data collected outside Fukushima Dai-ichi in April 2016, as presented in^26^.

**Based on the selection of parameter settings for the site “Stenungsund” in Sweden, as presented in^17^.

***Based on the upper end value of observed *D* and values in Sweden as presented in^17^.

*Approximating that the damping factor for the external dose contribution to a location inside a brick house will be the same as for a wooden house. This scales the dose values by the aforementioned *ESF* factor of 0.174 for brick houses and 0.345 for wooden houses (Table 1).

**Figure 6:**
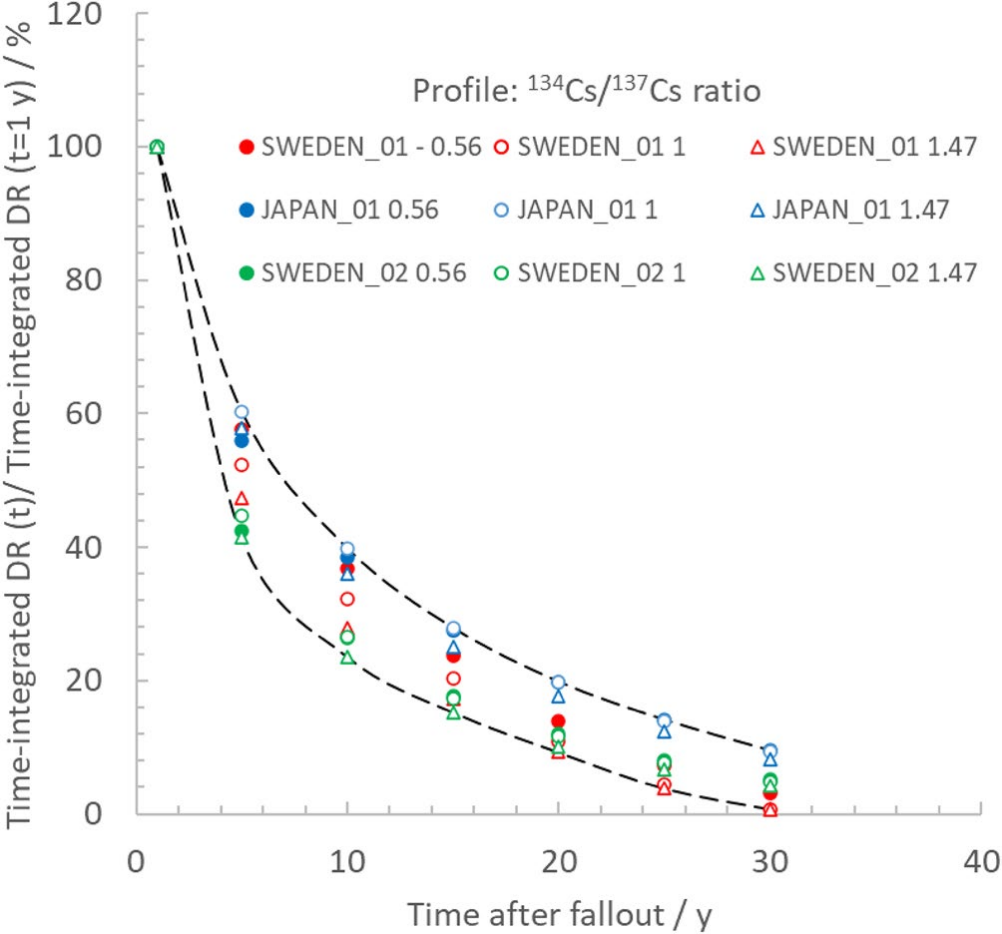
The action-influenced time-integrated dose reduction, *TDR*(*t*_*cleanup*_), in terms of averted effective dose by 5 cm soil removal, normalized to *TDR*(*t*_*cleanup*_ = 1 y) for the three different soil migration settings inTable 2 and three different ^134^Cs/^137^Cs initial isotope ratios at the time of fallout.

It can be estimated that short-lived fission products typically will add 15%–25% to the dose contribution from ^134^ and ^137^Cs during the first year after a NPP accident^1,11^, as observed in the radioactive fallout in Sweden after the Chernobyl accident and the Fukushima Northern trace. However, due to practical limitations, it may not be reasonable to fully implement and complete the cleanup operations until most of the short-lived fission products in the fallout have decayed. Hence, their contribution to the unmitigated dose has not been considered explicitly in the calculations. Therefore, it is more illustrative to compare the outcome of the cleanup operations with time accounting for what can ideally be obtained when starting 1 y after the fallout. Figure 6 illustrates the cumulative radiological benefit of topsoil removal as a function of time of cleanup, *t*_*cleanup*_, post-accident by plotting action-influenced time-integrated dose reduction, *TDR*, from ^134^Cs and ^137^Cs. The *TDR* values have been normalized to that for *t*_*cleanup*_ = 1 y. It is assumed that the residents have been evacuated in a non-affected zone upon return at *t*_*cleanup*_.

Figure 6 clearly shows that the *TDR* will gradually decrease with increasing *t*_*cleanup*_. If the topsoil removal measures are delayed for two decades, it will result in 10% or less in relation to the unmitigated effective dose when cumulated over 50 y post-fallout. This decrease is mainly an effect of that the resettling individual has spent an increasing time in a non-affected area before the return at *t*_*cleanup*_ in combination with the gradual decrease of the external dose rate from the ground deposition resulting from the migration of radiocesium in soil according to the settings in Table 2. The *TDR* vs. *t*_*cleanup*_ plot also shows that, for some soil migration parameters, very late soil removal efforts can be negligible, and can in theory even be negative due to tardy scraping effects. Note that the tardy scraping effect, in terms of an instantaneous increase in the effective dose, shown in the plots of Fig. 5, in theory could lead to a negative *TDR* for *t*_*cleanup*_ more than 20 y. However, for the studied soil settings the tardy scraping effects will only be transient shortly after *t*_*cleanup*_, and when the dose reduction, in terms of *TDR*, is integrated between *t*_*cleanup*_ and 50 y, this effect is no longer visible.

To put the averted doses from topsoil removal into context, it should be noted that for an average ground deposition for 1 MBq m^-2 137^Cs in the modelled neighborhood, the annual effective doses without remediation 1 y post fallout will be around 2 to 7 mSv y^-1^ depending on soil migration settings and the ^134^Cs/^137^Cs-ratio (including roof and street contribution which is not presented in this work). When a radiological and nuclear emergency phase is transgressed into an existing radiation exposure situation,^28^ suggests that a reference level for optimization of protective measures should be targeted towards the lower part of the interval between 1 to 20 mSv y^-1^. Preliminary calculations show that to achieve a dose rate below 1 mSv y^-1^ for a resettling evacuee in the modelled neighborhood after the cleanup at 5 y post fallout, would demand an *IDR* for soil removal of the garden areas of at least 0.3, and most likely a replacement of all contaminated roofs. A more exhaustive assessment of the dose contribution from the various types of surfaces in the modelled neighborhood is currently undergoing.

### Collective averted dose and generated soil waste per unit area

A cleanup operation of topsoil removal, ideally conducted 1 y after the fallout in a neighborhood consisting of wooden houses, will result in an individual averted effective dose for an adult ranging between 25 and 65 mSv per MBq m^-2 137^Cs^-1^ over 50 y after the fallout. The corresponding value for a neighborhood of brick houses will be 18 to 44 mSv per MBq m^-2 137^Cs. Assuming 2.8 residents per building in the neighborhood model by^19^, it will be then inhabited by (2.8$\times$15)/(19,600 m^2^) = 2140 individuals per km^2^ residential area if excluding service areas and highway construction not included in the model. The collective averted dose, obtained by multiplying the number of inhabitants per km^2^ with the averted dose for a single person for 5 cm topsoil removal at *t*_*cleanup*_ = 1 y will correspondingly range from 54 to 138 (manSv km^-2^)/(MBq m^-2^) ^137^Cs for a neighborhood of wooden houses and 27 to 69 manSv km^-2^ per MBq m^-2 137^Cs for a neighborhood of brick houses (Table 4). As a comparison, a corresponding calculation was performed for a multistory residential area with a population density of 11,600 inhabitants per km^2^, which is a typical number for a Swedish urban area^29^, and with a generic *ESF* set to 0.05, which is approximately the shielding factor presented for these types of buildings^30^. The values of these averted collective doses will essentially decrease with time according to Fig. 6, due to the same factors as for the individual residential doses.

**Table 3:** Unmitigated 50 y effective dose per unit ^137^Cs deposition, *E* (mSv/(MBq m^−2^ ^137^Cs) from residential garden surfaces for three types of soil migration settings (see Table 2), to residents in wooden or brick houses in the modeled urban area (excluding short-lived fission products) and the corresponding averted dose (integrated over 50 y) after a combination of evacuation prior to resettlement at time *t*_*cleanup*_ = 1 y and subsequent 5 cm topsoil removal from an NPP release with ^134^Cs/^137^Cs ratios of 0.56, 1, and 1.47. NC = Not computed.

	JAPAN_01			SWEDEN_01			SWEDEN_02		
	^134^Cs/^137^Cs-ratio								
	0.56	1	1.47	0.56	1	1.47	0.56	1	1.47
***Unmitigated dose***, (mSv/(MBq m^-2^)									
Indoor wooden house	65.9	67.9	79.0	58.9	71.3	86.2	34.2	36.5	43.9
Indoor brick house*	33.2	34.2	39.8	29.6	35.9	43.4	17.2	18.4	22.1
Free surface	238.2	277.1	318.6	244.5	289.2	337.0	133.3	161.7	192.0
***Unmitigated dose (1 y resettlement, no topsoil removal),*** (mSv/(MBq m^-2^)									
Indoor wooden house	47.6	53.4	60.6	47.0	54.1	63.3	21.6	24.8	29.1
Indoor brick house*	24.0	26.9	30.5	23.7	27.2	31.8	10.9	12.5	14.6
Free surface	206.1	236.3	256.6	207.9	236.3	266.7	106.3	122.5	139.8
Free surface									
***Averted dose (5 cm topsoil removal upon resettlement),*** (mSv/(MBq m^-2^)									
Indoor wooden house	47.6	53.4	60.6	47.0	54.1	63.3	21.2	24.1	28.3
Indoor brick house	24.0	26.9	30.5	23.7	27.2	31.8	10.7	12.1	14.2
Free surface	NC			207.9	236.2	266.3	NC

**Table 4:** Averted collective dose by evacuation and 5 cm topsoil removal at *t*_*cleanup*_ = 1 y for indoor occupancy per unit area and deposition of ^137^Cs (manSv km^-2^)/(MBq m^-2 137^Cs) in three typical residential areas for different ^134^Cs/^137^Cs ratios and for the three different soil migration parameters given in Table 2. Average population densities in one-story buildings and high-rise building areas are assumed to be 2140 inhabitants km^-2^ and 11,600 inhabitants km^-2^, respectively.

	Wood			Brick			Multistory		
^134^Cs/^137^Cs-ratio	0.56	1	1.47	0.56	1	1.47	0.56	1	1.47
JAPAN_01	126.3	121.0	134.3	63.6	60.9	67.6	99.1	95.0	105.4
SWEDEN_01	103.3	118.3	138.0	52.0	59.6	69.5	81.1	92.9	108.3
SWEDEN_02	54.5	57.4	62.0	28.9	27.4	31.2	42.8	45.1	48.7

Continued studies are needed to more precisely model how different soil migration parameters affect the fluence after cleanup in typical outdoor locations in residential and recreational areas. However, a rough approximation can be made using the corresponding averted dose for an infinite surface distribution of ^137^Cs migrating into standard soil (Table 3). For an outdoor occupancy factor of 20% (*f*_*out*_ = 0.2) in a typical Swedish soil migration setting (SWEDEN_01; Table 2), the averted individual dose for cleanup time, *t*_*cleanup*_ = 1 y, from a 5 cm topsoil removal would then range between 90 and 105 mSv per MBq m^-2 137^Cs. For a residential area consisting of one-story wooden houses, these values will correspond to an averted collective dose ranging from 90 to 105 (manSv km^-2^)/(MBq/m^-2^) ^137^Cs.

In addition to the benefits of topsoil removal in terms of averted radiation doses, such a measure also has negative side effects including the generation a waste and radiation exposures to cleanup workers. Hinrichsen et al.^31^ showed that, for a semi-urban area consisting of one-story residential buildings, 5 cm topsoil removal enclosing more than 12,000 m^2^ of the ground around a house (corresponding to 600 m^3^ of contaminated soil) would not significantly contribute to a further dose reduction inside a wooden or brick home. The study also showed that a dose reduction more than 80% in an urban area might be challenging to achieve if the large surrounding regions are not included in the topsoil removal. For the modelled suburban neighborhood used in this study (Fig. 1:Right), with a 19,600 m^2^ total surface, of which 15,400 m^2^ consist of garden surface, a 5 cm topsoil removal will generate at least 770 m^3^ of waste or 39$\times$10^3^ m^3^/km^2^ of such a neighborhood. Expressed in terms of waste per unit averted collective dose, assuming a neighborhood of wooden houses, a topsoil removal procedure would yield about 300–700 m^3^/(manSv)/(MBq m^-2^ ^137^Cs) depending on the soil migration settings. By computing the integral of the normalized concentration profile *C*_*norm*_(*x* = 5 cm, *t* = 1 y), and assuming a topsoil density ranging from 1 to 1.5 g cm^-3^, typical ^137^Cs activity concentrations in the removed soil layers 1 y after fallout are estimated to range between 8.3 and 13.0 (kBq kg^-1^)/(MBq m^-2 137^Cs) for the three types of soil migration settings. The corresponding values 5 y after the fallout range from 4.5 to 11.9 (kBq kg^-1^)/(MBq m^-2 137^Cs).

To summarize, a 5 cm topsoil removal before the return of evacuated residents will theoretically avert most of the projected 50 y effective dose (assuming more than 80% indoor occupancy after return) if conducted within a few years after the fallout. However, if delayed further, and when accounting for nonideal conditions when up to half of the topsoil activity content remains after cleanup^6^, deeper soil layers may need to be removed to achieve a > 90% dose reduction. However, deeper soil removal layers will also yield proportionally greater waste generation.

## Summary and conclusions

Based on previous Monte Carlo simulations and curve regressions for different soil burial depths, the theoretical action-influenced dose reduction from topsoil removal in residential areas consisting of one-story wooden or brick buildings was calculated for different soil removal depths, for three types of soils in terms of elemental cesium migration and diffusion rates and three different ^134^Cs/^137^Cs ratios in the initial NPP fallout.

The theoretical time-integrated dose reduction obtained from 5 cm topsoil removal of gardens in residential areas consisting of one-story buildings can be as high as 65% to 85% of the unmitigated 50 y integrated effective dose. In relation to the unmitigated dose, the averted doses will depend less on the initial ^134^Cs/^137^Cs ratio in the fallout and more on the soil-migration characteristics and the associated effective ecological half-time of the element. Furthermore, the 50 y time-integrated dose reduction will, in general, depend heavily on the timing of the cleanup operations, and if delayed more than two decades, the dose-rate reduction effect by topsoil removal will be negligible or even adverse due to the tardy scraping effect; i.e., the action uncovers radiocesium deposition in deeper layers and hence momentaneously increases the dose rate to the residents.

If conducted within the first year after the fallout, the averted collective doses by topsoil removal per unit decontaminated residential area will ideally be on the order of 100 (manSv km^-2^)/(MBq m^-2^) ^137^Cs), depending on soil migration settings and type of residential area. However, these collective doses are averted at the cost of waste generation on the order of 40$\times$10^3^ m^3^ or, as expressed in terms of waste per unit averted collective dose, more than 300 m^3^/(manSv)/(MBq m^-2^ ^137^Cs). Further investigation into the averted detriment through soil removal (and possibly other related decontamination procedures) should also include estimates of lifetime attributable risk based on different age and sex cohorts to understand how different generations benefit from these cleanup measures.

Further modeling and experimental studies could also be conducted on how the residual groundshine from decontaminated soil surfaces changes over time. The influence of sedimentation and erosion on the initial dose reduction and time-integrated dose reduction for topsoil removal could be modeled to understand their long-term influence on the dose rate from decontaminated soil surfaces.

## Supplementary Information

Supplementary Information.

## Data Availability

The datasets used and/or analysed during the current study available from the corresponding author on reasonable request.
