# Supplementary material for: Time-dependence of decontamination efficiency after a fallout of gamma-emitting radionuclides in suburban areas: a theoretical outlook on topsoil removal
Source: Sci Rep. 2022 Dec 15;12:21656. doi: 10.1038/s41598-022-25956-y (PMC9755301; doi:10.1038/s41598-022-25956-y)
Supplement: Supplementary file 1 — Supplementary Information. [file 41598_2022_25956_MOESM1_ESM.docx]

# Appendix 1

Consider a circular surface (Surface 1), with area *S_0_*, filled with a certain number of shielding objects that has been uniformly contaminated with a gamma emitter. The shielding objects (e.g. buildings) can be mimicked as an additional attenuating layer on top of the ground (See Fig. A.1). The gamma emitting deposition has in turn migrated into soil a certain penetration depth *x*. The air kerma rate 1 m above ground at the center of this surface is *z*. Consider now a similar surface (Surface 2) of similar size, with the same amount of surface deposition density as the previous surface but with no ground penetration. The corresponding air kerma rate in the middle of Surface 2 is denoted *q*. The relative damping factor, *RDF*(x), for this geometry and ground penetration, *x*, will then be formed by the ratio between these two air kerma rates (A.1):

|  | *RDF*(*x*, *S_0_*)=*z*(*x*, *S_0_*))/*q*(*x*=0, *S_0_*) | (A.1) |
| --- | --- | --- |

If we now expand the considered areas, from size S0 to size S0+ΔS for the two surfaces (Surface 1 and Surface 2), and assume that the expanded surfaces have the same surface deposition density of the gamma emitter as the Surfaces 1 and 2, the gamma emitters in the expanded surfaces will contribute with an air kerma rate of *p* to the air kerma rate in the middle of Surface 1, giving a summed air kerma rate for the expanded Surface 1 of *z*+*p*. The corresponding additional contribution of air kerma rate for Surface 2, however, will be larger than *p*, here denoted as the product *a***p*. The value of *a* will depend on the relative increase in expanded surface, ΔS, and the average amount of shielding objects per unit area in the considered surfaces. *RDF* for the expanded surface will be (A.2):

|  | *RDF*(*x*, *S_0_+*Δ*S*)=(*p*+*z*(*x*, *S_0_*))/(*a*⋅*p*+*q*(*x*, *S_0_*)) | (A.2) |
| --- | --- | --- |

As can be visualized in Fig. A.1, the attenuation of the shielding objects, represented as a layer with a certain density *ρ*, will be roughly the same for Surface 1 and Surface 2 (almost the same spectrum penetration length in the radiation path), whereas in Surface 1 there will be an additional attenuation by the soil in which the gamma emitter is buried. Thus, for the expanded considered surface, the contribution from the non-penetrated surface contamination to the air kerma rate in the middle of Surface 2, will always be larger than the corresponding contribution from the expanded surface for a source with burial depth x, meaning that *a* will always be larger than 1. Consequently, for a given penetration depth in Surface 1, the *RDF*-function in Eq. A.2 will monotonously decrease with increasing considered area, *S_0_*+Δ*S*.


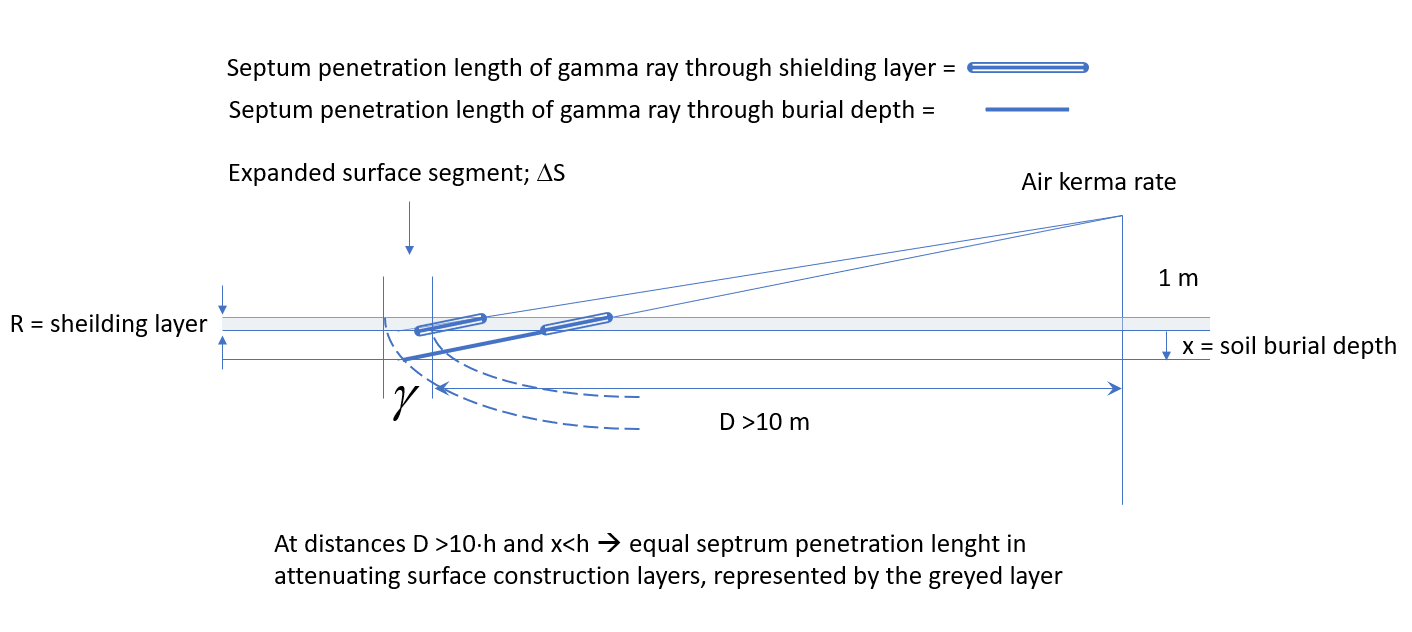


**Figure A.1.** Irradiation geometry of an area containing shielding objects that are represented by an attenuating layer with density ρ and thickness R.
